# Antimicrobial, antibiofilm, DNA binding, and antioxidant activities of green synthesized zinc oxide nanoparticles using the wild mushroom *Lepista sordida* (Schumach) singer EGDA2

**DOI:** 10.1186/s12934-026-02952-9

**Published:** 2026-03-12

**Authors:** Amira A. El-Fallal, Mahmoud M. Nour El-Dein, Shimaa R. El-Esseily, Nancy M. Mansour, Mohamed M. El-Zahed

**Affiliations:** 1 Botany and Microbiology Department, Faculty of Science, Damietta University, P.O. Box: 34517, New Damietta, 34517 Egypt; 2 Chemistry Department, Faculty of Science, Damietta University, Damietta, Egypt

**Keywords:** *Lepista sordida*, Green synthesis, ZnO, Antimicrobial, Antibiofilm, DNA binding, Antioxidant

## Abstract

**Background:**

Microbial resistance is a major public health concern, leading to treatment failure, prolonged hospitalization, and increased mortality. Developing new, cost-effective antimicrobial agents is critical. This study presents a simple, economical, and eco-friendly (green) synthesis of zinc oxide nanoparticles (ZnO NPs) using bioactive metabolites from the mushroom *Lepista sordida* EGDA2 as a bio-nano-factory. The resulting ZnO NPs were characterized by ultraviolet–visible spectroscopy (UV–Vis), X-ray diffraction (XRD), Fourier transform infrared spectroscopy (FTIR), transmission electron microscopy (TEM), and zeta potential analysis, while the bioactive capping agents were profiled using GC–MS. UV–Vis analysis confirmed ZnO NP formation with a characteristic absorption peak at 301 nm, and XRD and TEM revealed crystalline wurtzite-structure nanoparticles (NPs) with an average size of approximately 33 nm (18–46 nm). FTIR and GC–MS indicated the presence of stabilizing bioactive compounds, notably fatty acids (e.g., palmitic acid, oleic acid) and polyphenols, and the NPs exhibited good colloidal stability, with a zeta potential of − 21.83 ± 4.25 mV. The biosynthesized ZnO NPs showed potent, dose-dependent antimicrobial and antibiofilm activities against a panel of drug-resistant bacteria (e.g., MRSA and *Pseudomonas aeruginosa*) and pathogenic fungi (e.g., *Candida albicans* and *Aspergillus niger*), with minimum microbicidal concentration (MMC) values correlating with minimum inhibitory concentration (MIC) values and up to 70% inhibition of biofilm formation in MRSA and *P. aeruginosa* at 150 µg/mL, consistent with the reported antibiofilm potential of green-synthesized ZnO NPs. The NPs also exhibited strong antioxidant activity in the DPPH assay (78.1% inhibition) and interacted with calf thymus DNA (CT-DNA) via an intercalative binding mode, in line with previous reports on ZnO NP–DNA interactions. Cytotoxicity assessment using the MTT assay on Vero cells yielded a high CC_50_ value of 208.17 ± 1.94 µg/mL, indicating that the biogenic ZnO NPs are biocompatible at their effective antimicrobial and antibiofilm concentrations (≤ 150 µg/mL), which agrees with the generally favorable biocompatibility profile reported for green-synthesized ZnO NPs on Vero cells.

**Conclusions:**

This work presents a promising, safe, and effective alternative treatment utilizing green synthesized ZnO NPs capped with bioactive compounds from *L. sordida* EGDA2. The resulting NPs demonstrate synergistic antioxidant, antimicrobial, and significant antibiofilm activities, offering promising applications in industrial, pharmaceutical, and environmental applications.

**Graphical abstract:**

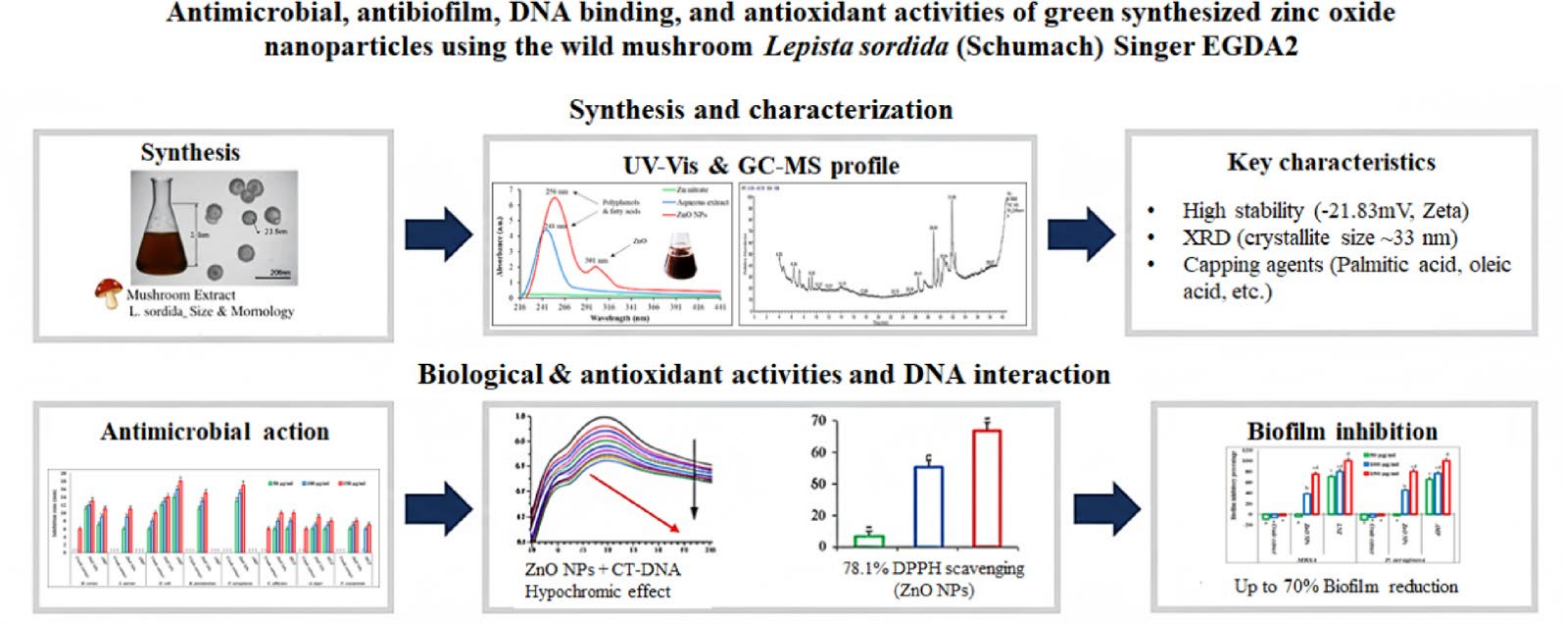

## Background

The purple basidiomycete, *Lepista sordida*, an edible and therapeutic fungus belonging to the family Tricholomataceae, is a promising source of pharmacologically active compounds [1, 2]. *L. sordida* demonstrates significant nutritional value, and contains a diverse array of bioactive metabolites, while maintaining an appealing taste profile [3, 4]. Several investigations have explored the pharmacologically active compounds present in *L. sordida* fruiting bodies for potential medical applications. Multiple bioactive metabolites have been successfully extracted and characterized from *L. sordida* cultures, including terpenoids (lepistal and lepistol), sesquiterpenes, exopolysaccharides, diatretol, lepistamides, and diketopiperazines [5]. These compounds demonstrate diverse biological activities, including antitumor, anti-aging, antibacterial, antifungal, antioxidant, immunoregulatory, and antimicrobial effects [6, 7]. Notably, Chen et al. [8]. demonstrated that lepistamides A–C and 3, 6-dioxygenated diketopiperazines possess antimicrobial activity, showing particular efficacy against *Staphylococcus aureus*.

On the other hand, microbial resistance represents a major disadvantage, driving the urgent need for novel antimicrobial agents, as this issue poses a serious threat to human health [9]. When infections are caused by antibiotic-resistant microorganisms, both the cost and time of treatment increase. The extensive abuse of antibiotics and other antimicrobial agents has led to an increase in the number of resistant microbes [10]. Numerous fungi, including *Aspergills niger*, *Penicillium* sp., and *Fusarium oxysporum*, as well as bacteria including *S. aureus*, *Escherichia coli*, *Klebsiella pneumoniae*, *Salmonella* sp., and *Pseudomonas aeruginosa* have been found to be resistant to current and commercial antibiotics and antifungal agents [10, 11]. Therefore, alternative antimicrobial agents must be obtained in order to address the growing issue of microbial resistance.

Recently, nanomaterials have been used as potent antibacterial, antifungal, antiprotozoal, antilarval, and antiviral agents in a variety of pharmaceutical, agricultural, and industrial applications due to their high stability, and distinguished antimicrobial potential [12–14]. To increase their antimicrobial action, they were added and mixed with various substances [15, 16]. The current state-of-the-art in combating multidrug-resistant pathogens involves developing novel materials, including metal oxide nanoparticles (NPs), which offer distinct mechanisms of action compared to conventional antibiotics. Among these, zinc oxide nanoparticles (ZnO NPs) have emerged as particularly promising candidates due to their strong, broad-spectrum antimicrobial activity (against bacteria, fungi, and yeast), low intrinsic toxicity to mammalian cells (compared to certain other metals), and cost-effectiveness in large-scale synthesis. Due to zinc ions’ interaction with biological macromolecules, they can harm the cell wall of microbial cells, stop the production of new cells, limit protein synthesis, decrease membrane permeability, and impede microbial metabolic processes [17]. The distinct chemical, physical, and biological characteristics of ZnO NPs—such as their mechanical, magnetic, piezoelectric, strong adsorption, high catalytic efficiency, biocompatibility, high isoelectric point, fast electron transfer kinetics, solubility, and cellular uptake—make them more reactive and give them a stronger antibacterial effect than bulk Zn [18, 19]. ZnO NPs are distinguished from their bulk counterparts by their special characteristics, which include their extremely small size, high surface area -to- volume ratio, and strong oxidative effects. Furthermore, studies have revealed that inorganic NPs are smaller particles with a vast surface area that allow them to readily penetrate cells through tiny gaps in plasma membranes [20]. ZnO NPs exert their antimicrobial effect mainly through the generation of reactive oxygen species (ROS) and the release of Zn^2+^ ions, which together promote cell wall and membrane damage, enzyme inhibition, and oxidative stress, ultimately leading to microbial cell death [21–23].

The NPs were synthesized using a variety of methods, including chemical, biological, and physical methods. Due to the high temperature and pressure required for physical processes, specialized equipment is required. Since chemical methods need capping agents to stabilize the NPs, they have the advantage of creating large quantities of NPs quickly, but they can result in hazardous and unfavorable byproducts [24]. These days, green-manufactured nanoparticles have garnered a lot of attention because of their exceptional biocompatibility and environmentally beneficial characteristics. Reducing or eliminating hazardous substrates is the goal of green practices. However, the use of hazardous chemicals is not required for biological approaches to NP synthesis. According to Majeed et al. [25]. , the biological production of NPs is a single-step bio-reduction process that requires less energy and yields environmentally favorable outcomes. In particular, biological molecules can reduce the harmful qualities generated by the typically integrated NPs and act as capping agents. Plants and microorganisms, such as bacteria and fungi, are used to create NPs in an environmentally friendly way [26]. In contrast to intracellular biosynthesis, which necessitates the extraction and purification of NPs from plant or microbial cultures, extracellular biosynthesis of NPs is given more priority [27]. Despite the promise of biogenic ZnO NPs, a significant knowledge gap remains in utilizing and characterizing extracts from less-studied higher basidiomycete fungi for NPs functionalization. Most green syntheses rely on plant extracts, leaving the unique chemical profiles of medicinal fungi largely unexplored in nanobiotechnology. In an extracellular model, herein, the fruiting body extract of *L. sordida* EGDA2 (AC: LN827702) was used as a bio-reducing agent to create ZnO NPs, taking advantage of all the benefits of *L. sordida* and its potent active bio-compounds. To the best of our knowledge, the present work is the first to report a simple, rapid, cost-effective, and environmentally friendly biosynthesis of ZnO NPs using an aqueous extract from the fruiting body of *L. sordida*. This approach eliminates the need for complex and specialized procedures such as isolation, culture maintenance, and multiple purification steps.

Surface functionalization is a critical process in nanotechnology that involves deliberately modifying the outermost layer of NPs to enhance or introduce specific properties required for targeted applications, particularly in biomedicine [28, 29]. The green-synthesis of ZnO NPs using *L. sordida* occurs naturally through the biomolecules present in the *L. sordida* extract such as polyphenols, fatty acids, and proteins [30]. These biomolecules play key roles in enhancing stability and biocompatibility—(the attached organic layer acts as a capping and stabilizing agent, preventing particle aggregation and potentially reducing non-specific protein binding. Consequently, this improves the colloidal stability and biocompatibility of the NPs within biological systems. Moreover, surface functionalization governs the interaction of NPs with biological targets [31].

The bioactive compounds from the mushroom extract, once anchored on the NPs surface, confer new or enhanced properties, such as improving antimicrobial efficacy by increasing membrane affinity and potentially synergizing with the ROS generation of the ZnO core [32]. Therefore, the surface functionalization of ZnO NPs with *L. sordida* extract is not merely a byproduct of synthesis; it represents a deliberate strategy to obtain a hybrid nanomaterial with synergistic therapeutic potential for biomedical applications, combining the intrinsic antimicrobial strength of ZnO with the bioactive benefits of the mushroom constituents.

Accordingly, the antimicrobial properties of the biosynthesized ZnO NPs were evaluated against several Gram-positive and Gram-negative bacterial strains, as well as fungal pathogens, together with their antibiofilm, DNA binding, and antioxidant activities. Utilizing bioactive compounds from the mushroom *L. sordida* EGDA2 not only provides a non-toxic, green synthesis route but also transfers the antioxidant and antimicrobial properties of the capping agents directly to the NPs surface, thereby enhancing the overall bioactivity of the formulation.

## Materials and methods

### Microbial strains

The fungal and bacterial strains utilized for the antimicrobial activity or the green synthesis of ZnO NPs were graciously provided by the Microbiology Laboratory, Faculty of Science, Damietta University, Egypt. Dry fruiting bodies of wild-grown *L. sordida* (Schumach) Singer EGDA2 (dried at 45 °C for 72 h) were collected from El-Senania lemon fruit farms at N 31.4403 °, E 31.7776 ° (Damietta, Egypt). Its identification was performed in accordance to Singer [33] and confirmed by the ITS DNA sequencing. It was deposited in the database with accession number LN827702 [34].

### Preparation of the aqueous mushroom extract

The fruiting bodies were air dried, the mushroom samples were cut into small pieces and milled to fine powder, which was then mixed with 100 mL of hot distilled water (60 °C) for 10 min. Then, it was re-stirred for 60 min at the room temperature (25 °C), centrifuged at 4000 rpm for 30 min to remove the insoluble substances, and finally the aqueous supernatant was collected. The residue was then extracted again using the same procedure [35]. Whatman filter paper No. 1 was used to filter the aqueous mushroom extract, which was then kept at 4 °C.

### Green synthesis of ZnO NPs

Zinc nitrate hexahydrate (Zn (NO_3_)_2_.6H_2_O, crystallized, ≥ 99.0%, Sigma- Aldrich) was prepared as a 1 mM solution for green synthesis purposes to serve as the primary source of zinc ions (Zn^2+^). This solution (40 mL) was combined with 20 mL of aqueous mushroom extract (2:1 v/v ratio) and incubated in an orbital shaker for 72 h at 120 rpm and 30 °C in the dark. A color change to turbid yellowish-white indicated the potential formation of ZnO NPs. The formation of ZnO NPs was further confirmed by characterizing them using various analytical methods [36].

### Characterization of ZnO NPs

The biosynthesized ZnO NPs were characterized using the following techniques. The evolution of the reaction between the metal ions and the mushroom extract was observed using UV-visible spectra (UV-Vis) of ZnO NPs in aqueous solution. The chemical functionalized groups present and associated with the green synthesized ZnO NPs were investigated using Fourier Transform Infrared Spectroscopy (FTIR). The UV-Vis absorption (Double-beam spectrum UV-Vis spectrophotometer V-760, JASCO, UK) and FTIR (FT/IR-4000 Series, JASCO, UK) services were rendered by the Central Lab of the Chemistry Department of Damietta University in Egypt. The charge of ZnO NPs was measured at Damietta University in Egypt’s Center for Excellence in Research of Advanced Agricultural Sciences (CERAAS) using the Nano-ZS90 Zetasizer (Malvern, UK). Using a Transmission electron microscopy (TEM, JEM-2100, JEOL, Japan) adjusted to 180 KV, the size and surface morphology of ZnO NPs were evaluated. Genesis Energy dispersive X-ray (EDX) elemental analysis system was used to investigate the elemental composition and to generate the elemental mapping profile of the ZnO NPs. The composition and structure of the pure ZnO NPs were examined using X-ray diffraction (XRD, Bruker D8 Discover Cu target wavelength 1.54 Å, 40 kV, and 40 mA, Germany) at the Central Metallurgical Research and Development Institute (CMRDI), Cairo, Egypt.

### Gas chromatography–mass spectrometry (GC–MS) analysis

A Trace GC-TSQ mass spectrometer (Thermo Scientific, Austin, TX, USA) and a direct capillary column TG–5MS (30 m × 0.25 mm × 0.25 μm film thickness) were used to determine the chemical composition of ZnO NPs coated with mushroom bioactive compounds. The column oven’s temperature was initially maintained at 50 °C, then increased at 5 °C per minute to 250 °C, where it was held for two minutes. The temperature was then ramped to 300 °C at 30 °C per minute and held for two minutes. Helium was used as a carrier gas at a constant flow rate of 1 mL per minute, and the injector and MS transfer line temperatures were maintained at 270 and 260 °C, respectively. Diluted samples of 1 µL were automatically injected using the Autosampler AS1300, coupled with GC in split mode, following a 4-minute solvent delay. EI mass spectra were acquired in full scan mode at 70 eV ionization voltage over the m/z range 50–650. The ion source temperature was set at 200 °C. Components were identified by comparing their mass spectra with those in the WILEY 09 and NIST 14 mass spectral databases [37].

### Antimicrobial potential of ZnO NPs using the agar well diffusion test

Fungal strains (*Aspergillus niger* van Tiegh, *F. oxysporum* f. sp. *lycopersici* Fol4287), Gram-positive bacteria (*Bacillus cereus* ATCC 14579 and methicillin-resistant *S. aureus* ATCC 43300; MRSA), and Gram-negative bacteria (*Escherichia coli* ATCC 25922, *K. pneumoniae* ATCC 33495 and *Pseudomonas aeruginosa* ATCC 27853), and yeast (*Candida albicans* ATCC 10231) were used to study the ZnO NPs’ antimicrobial activity. Fungal and bacterial cells were grown at 30 °C in DOX broth medium for five days or 37 °C in nutrient broth medium for 24 h; then, cells with 1 × 10^6^ colony-forming units/mL (CFU/mL) or 1 × 10^8^ CFU/mL were cultured on new DOX or nutrient agar media for fungi and bacteria, respectively. The agar well diffusion method was used [38]. 100 µL of various ZnO concentrations (50, 100, and 150 µg/mL) were prepared and tested. Ampicillin (AMP) and miconazole (MCZ) were used as positive controls. Plates were incubated at 30 °C for five days or 37 °C for 24 h for fungi and bacteria, respectively. After incubation, zones of inhibition (ZOI) were recorded in mm.

### Minimum Inhibition concentration (MIC)

ZnO NPs’ MIC values against MRSA, *P. aeruginosa*, and *C. albicans* were evaluated using the broth dilution technique [39, 40]. The tested strains were added to broth media that had been treated with 0–150 µg/mL of the tested NPs. The mixture was then incubated for 24 h at 150 rpm and 37 °C for bacteria or 30 °C for yeast, respectively. A spectrophotometer was used to measure the growth rate at 600 nm.

### Minimum microbicidal concentration (MMC)

Using the pour plate approach, 50 µL aliquots from each MIC that displayed no obvious microbial growth were seeded onto the agar plates. Following this, the plates were incubated for 24 h at either 37 °C for bacteria or 30 °C for yeast. After assessing the growth of microbial colony, the CFU/mL was counted to determine the MMC values [41, 42].

### Antibiofilm test

ZnO NPs’ antibiofilm potential against MRSA and *P. aeruginosa* was examined by inoculating 96-well polystyrene titer plate wells with 100 µL of glucose-TSB (1% w/v) and 10 µL of bacterial solution (0.5 MacFarland). Following a separate addition of ZnO NPs (50, 100, and 150 µg/mL) to the wells, they were incubated at 37 °C for 24 h. Controls were also prepared, media with a bacterial suspension in the positive control well, and sterile medium only in the negative control well. After the cultures were removed from the incubator, the wells were washed with sterile distilled water. The remaining biofilm was stained with 0.25% crystal violet for 20 min. After staining period, the plates were gently washed three times with sterile distilled water and allowed to air dry. To quantify the biofilm, 200 µL of 33% acetic acid was added to each well to solubilize the crystal violet bound to the adhering cells. The absorbance of the resulting solubilized dye was then measured at 570 nm using a spectrophotometer [14]. Linezolid (LNZ) and AMP were used as standard antibiofilm agents against MRSA and *P. aeruginosa*, respectively.

### DNA binding test

The binding characteristics of ZnO NPs to calf thymus DNA (CT-DNA) were examined using electronic absorption spectrometry [43]. The intrinsic binding constant (K_b_) of the chemical was measured using calf thymus DNA using the formula: $\frac{{\left[ {{\mathrm{DNA}}} \right]}}{{\left( {{\varepsilon _{\mathrm{a}}} - {\varepsilon _{\mathrm{f}}}} \right)}}=\frac{{\left[ {{\mathrm{DNA}}} \right]}}{{\left( {{\varepsilon _{\mathrm{b}}} - {\varepsilon _{\mathrm{f}}}} \right)}}+\frac{1}{{{K_{\mathrm{b}}}\left( {{\varepsilon _{\mathrm{a}}} - {\varepsilon _{\mathrm{f}}}} \right)}}$

where є_a_ is the molecular extinction coefficient recorded for the A_obs_/[ZnO NPs] at the chosen DNA concentration, є_f_ is the molar extinction coefficient of the ZnO NPs in solution, є_b_ is the molar extinction coefficient of the ZnO NPs when fully bonded to DNA, and [DNA] is the base pair concentration of calf thymus DNA. In p1ots of [DNA]/(є_a_–є_f_ ) versus [DNA] by slope to intercept ratio, K_b_ is represented.

### Antioxidant assay

According to Das et al. [44]. , the antioxidant and DPPH free radical scavenging activity of ZnO NPs were examined. Ascorbic acid was used as a standard antioxidant agent to compare the results. The samples and standards received varying concentrations of the DPPH solution (5–100 µg/mL) independently. Before measuring the absorbance readings, samples were incubated at 25 °C for 30 min. The inhibition of DPPH (I%) was calculated using the formula I*% = (A*_*control*_ *– A*_*compound*_*/A*_*control*_*) × 100*, where *A*_*control*_ represent the optical density in the absence of the compound and *A*_*compound*_ represents the optical density in its presence.

### Cytotoxicity assay

The cytotoxicity of green-synthesized ZnO NPs was evaluated against Vero cells (ATCC, Rockville, MD) using the MTT colorimetric assay according to the protocol described by Mosmann [45]. Cells were maintained in Dulbecco’s Modified Eagle’s Medium (DMEM) supplemented with 10% heat-inactivated fetal bovine serum, 1% L-glutamine, and 50 µg/mL gentamicin. Briefly, cells were seeded in 96-well plates at a density of 1 × 10^4^ cells/well and incubated at 37 °C in a humidified atmosphere 5% CO_2_ for 24 h to allow cell attachment. Subsequently, the cells were treated with varying concentrations of ZnO NPs (0–500 µg/mL) for a further 24 h. After treatment, cell viability was quantified based on the reduction of MTT to formazan crystals. Roflumilast was used as a standard anti-inflammatory agent for comparison. Dose-response curves were generated by plotting cell survival percentages against NPconcentrations to determine the inhibitory effects.

### Statistical analysis

The mean of three replicates (*n* = 3) ± standard deviation (SD) was used for all reported data. The calculation of the mean and SD, as well as the generation of the final graphs were performed using Microsoft Excel 356. A one-way ANOVA was used to compare the results at the 5% level of significance (*p* < 0.05), followed by a least significant difference (LSD) post hoc test.

## Results and discussion

Edible mushrooms such as *Agaricus* sp., *Coprinus* sp., *Pleurotus* sp., and others have recently attracted considerable attention as promising biological precursors for synthesizing bioactive metal NPs [46]. The interaction, fate, and eventual desired or adverse outcomes of nanomaterials in biological systems are fundamentally governed by a complex interplay of their characteristic parameters. These parameters can be categorized as intrinsic (inherent physicochemical properties) and extrinsic (surface properties influenced by the environment or synthesis conditions) [25]. Intrinsic parameters such as shape, size, crystal structure, and chemical composition—directly influence the specific surface area, dissolution rate (e.g., Zn^2+^ release from ZnO), and quantum effects. Particle size, for example, dictates cellular uptake mechanisms (endocytosis vs. passive diffusion) as well as clearance rates from the body [47]. In contrast, extrinsic parameters—such as surface charge (Zeta potential), surface coating, agglomeration state, and surface energy—govern the formation of the biomolecular corona upon contact with biological fluids and mediate electrostatic interactions with cell membranes and proteins. These properties affect particle stability and toxicity, help prevent agglomeration, and impart enhanced functionality and overall stability [48, 49].

**Fig. 1:**
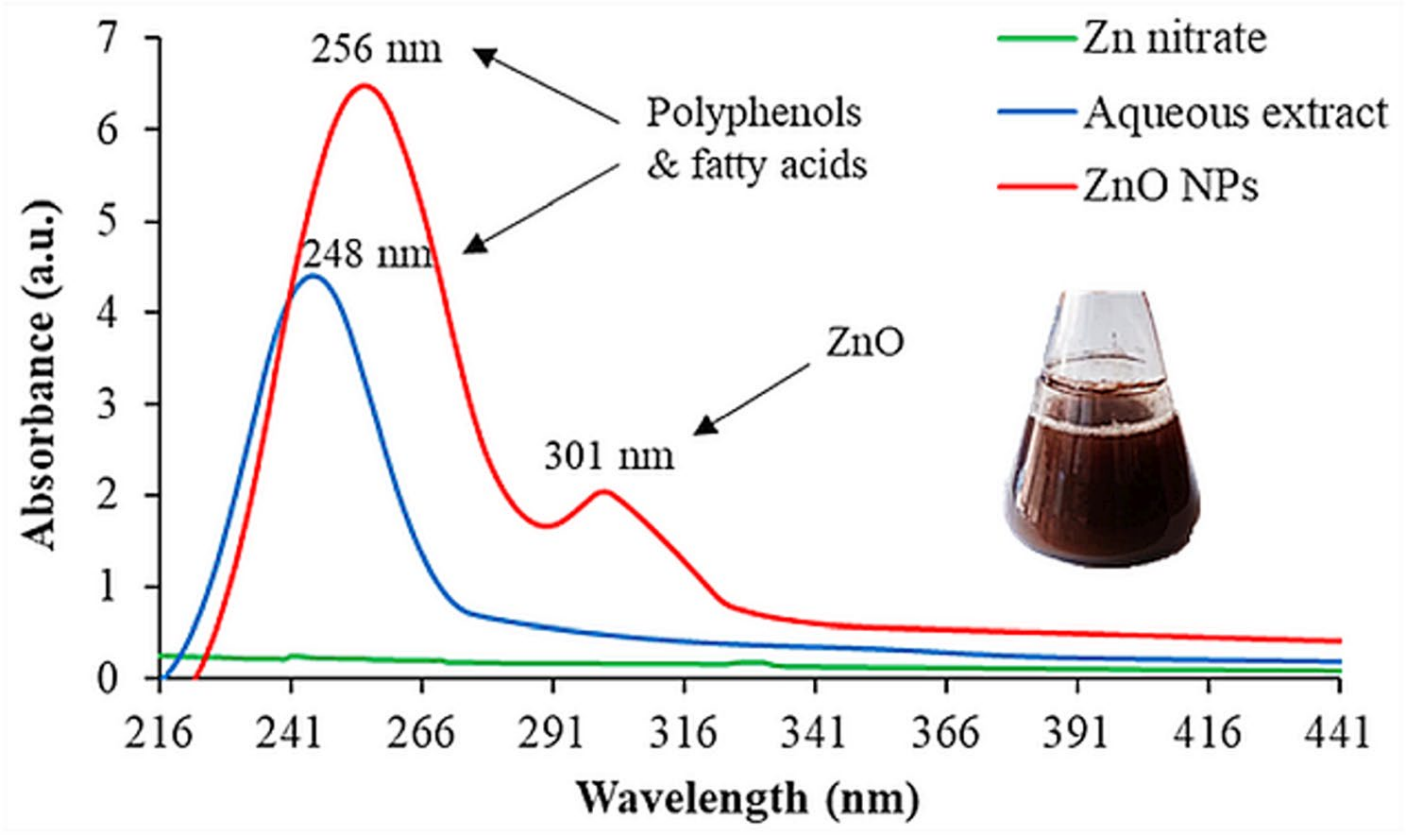
UV-Vis spectrum of the green synthesized ZnO NPs colloidal solution using *L. sordida* EGDA2 compared to the bulk solution of Zn (NO_3_)_2_.6H_2_O and the control aqueous extract

To confirm and characterize the formation, structural properties, morphology, and stability of the green synthesized ZnO NPs, several analytical techniques can be employed. In the present study, the formation of ZnO NPs using *L. sordida* EGDA2 was initially indicated by a visible color change of the reaction mixture-form pale brown at the start of the experiment to a turbid brownish white after incubation period (Fig. 1). This transformation is attributed to the mushroom extract’s rich composition of natural reducing and stabilizing agents, which not only facilitate NPs formation but also enhance stability. The surface plasmon vibrations of the activated ZnO NPs were responsible for the sample’s turbid brownish white hue [50]. The distinctive absorption peak of ZnO NPs, which is usually found at 301 nm for ZnO NPs, was used as a reference point in UV-Vis spectroscopy to initially establish the production of the ZnO (Fig. 1) [51]. The bandgap of ZnO, a semiconductor, is broad (around 3.37 eV). When its size is reduced to the nanoscale, electrons and holes are confined within a limited volume, increasing the NPs’ effective bandgap energy. A decrease in wavelength (*λ*) is equivalent to an increase in energy (*E*) because energy and wavelength are inversely related (*E = hc/λ*; *h* is Planck’s constant; *c* is the speed of light in a vacuum) [52]. Consequently, the absorption peak of ZnO particles shifts to a shorter wavelength (a blue shift) as their size decreases, which is commonly observed when the NPs are capped with polymers [53]. In the control aqueous extract of *L. sordida* EGDA2, the sample’s color remained unchanged, suggesting that the only substances that produced ZnO NPs were phyto-bioactive chemicals and mushroom extract. The presence of polyphenols and fatty acids in the crude extract of *L. sordida* EGDA2 may be the cause of other peaks in the UV-Vis spectra of aqueous extract of *L. sordida* EGDA2 and ZnO NPs that emerged at 248 nm and 256 nm, respectively [54]. These results are consistent with those of Zhao et al. [55]. who reported fatty acid peaks at 286 and 291 nm. The observed spectral shifts suggest potential molecular interactions and complex formation among the constituents, reflecting changes in the electronic environment of the ZnO NPs.

**Fig. 2:**
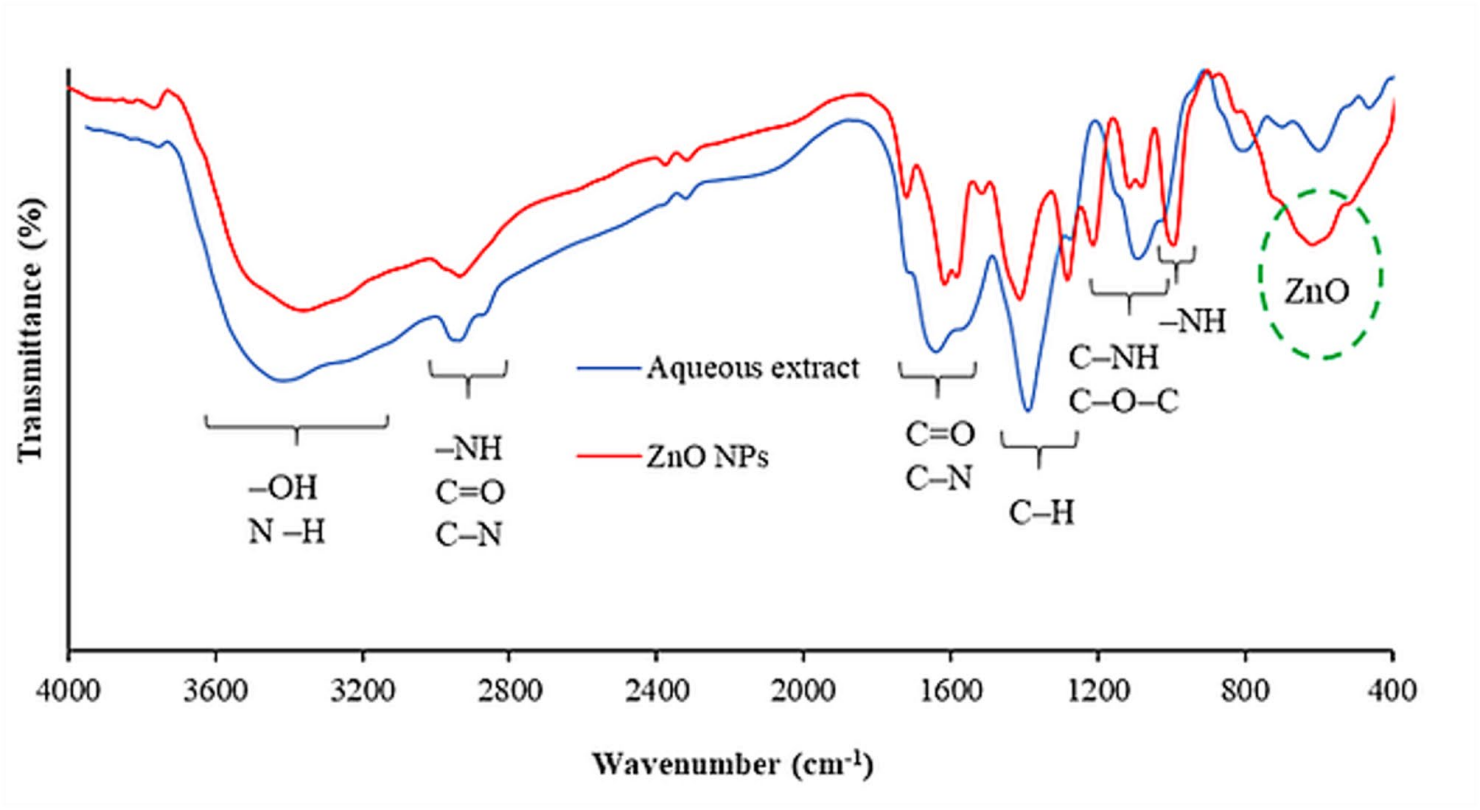
FTIR spectra of ZnO NPs and the aqueous extract of *L. sordida* EGDA2

The ZnO NPs colloidal solution was analyzed using FTIR, as shown in Fig. 2. The FTIR spectrum of the NPs shows several characteristic absorption bands: broad bands at 3200–3300 cm⁻¹ correspond to O–H and N–H, while peaks around 400–800 cm⁻¹ indicate Zn–O stretching vibrations. The C = O stretching near 2928, 1628, and 1582 cm⁻¹, as well as bands between 979 and 1118 cm⁻¹, reflect C–O–C and C–NH stretching, confirming successful integration and molecular interaction between ZnO and the mushroom bioactive compounds. The method made it possible to identify important functional groups like hydroxyl (–OH), amino (–NH₂), ester (C–O–C), and carbonyl (C = O), which confirmed the presence and successful incorporation of proteins, polyphenols, and fatty acids. Other bands at 1600–1500 cm⁻¹ indicate C–N bending and C–H deformations, which further support interactions with ZnO NPs and embedded molecules. Peaks in the 1002–1079 cm⁻¹ range correspond to C–O–C and C–NH stretching. Additionally, shifts in characteristic absorption bands provided evidence of hydrogen-bonding interactions between ZnO NPs and the organic components, indicating strong interfacial bonding and enhanced structural stability of the composite. Most importantly, the distinct band at ~ 570 cm⁻¹ confirms the presence of Zn–O vibrations, confirming retention of ZnO within the composite [56]. Collectively, the spectral shifts and intensity variations reveal the formation of coordination and hydrogen bonds between ZnO NPs and the organic matrix, resulting in a stable, well-integrated nanocomposite.

**Fig. 3:**
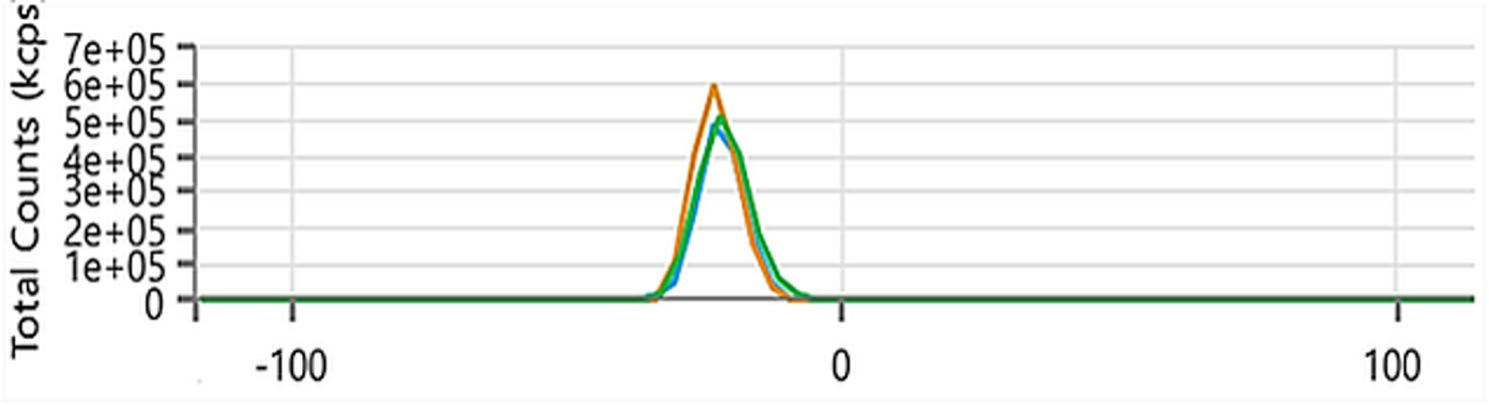
Zeta potential distribution of ZnO NPs

The zeta potential measurement of the synthesized ZnO NPs, as illustrated in Fig. 3, revealed a negative surface charge of − 21.83 ± 4.25 mV, indicating strong electrostatic repulsion between particles and good colloidal stability. This negative charge is typically attributed to the surface hydroxyl groups present on ZnO NPs [57]. Such a surface potential not only enhances the stability of the NPs but may also promote favorable electrostatic interactions with negatively charged bacterial membranes, thereby contributing to the antimicrobial and antioxidant activities of ZnO NPs [58].

**Fig. 4:**
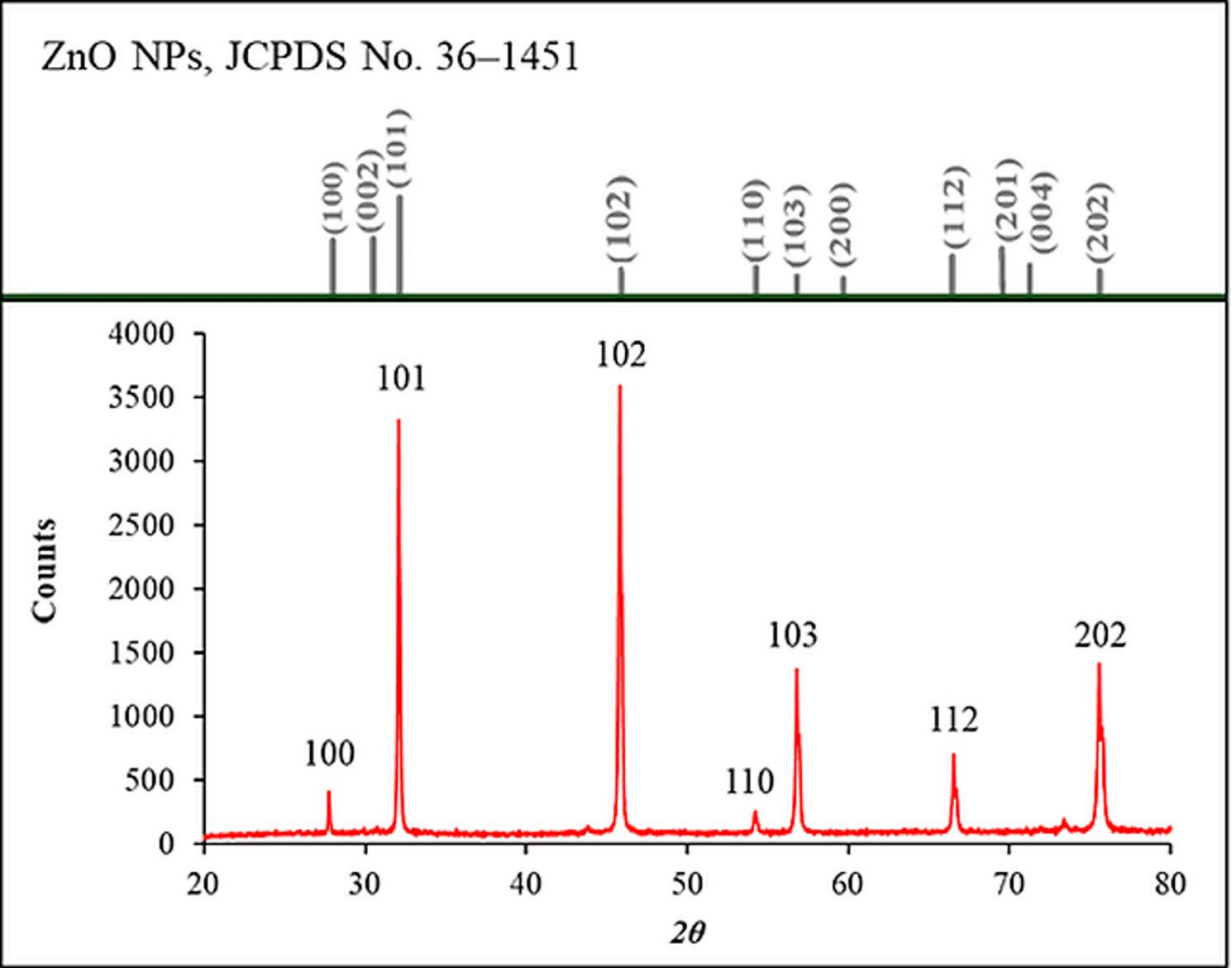
XRD patterns of the synthesized ZnO NPs

According to the JCPDS card no. 36-1451 (Fig. 4), the XRD pattern of the synthesized pure ZnO NPs displayed distinct and sharp diffraction peaks at 2*θ* values of 27.8°, 32.1°, 45.8°, 54.2°, 56.8°, 66.5°, and 75.6°, which correspond to the (100), (101), (102), (110), (103), (112), and (202) planes of the wurtzite crystalline structure. The presence of these characteristic peaks confirms the successful synthesis of phase-pure and highly crystalline ZnO NPs. The average crystallite size was estimated to be ~ 33 nm using the Scherrer equation, *D = Kλ/ (β cos θ)*, where K is the shape factor, λ is the X-ray wavelength, β is the full width at half maximum (FWHM), and *θ* is the Bragg diffraction angle.

**Fig. 5:**
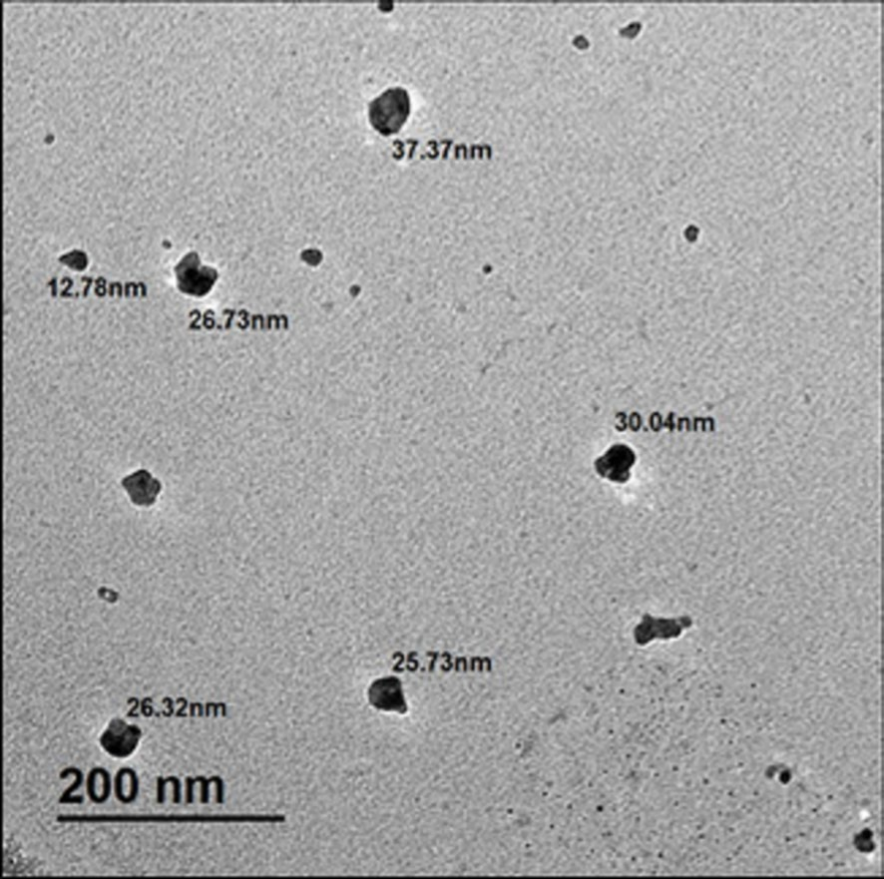
TEM images of ZnO NPs with scale bar = 200 nm

The green-synthesized ZnO NPs were found to be well-dispersed and quasi-spherical NPs with smooth edges, according to the TEM investigation (Fig. 5). The measured particle diameters, which ranged from approximately 12.78 nm to 37.37 nm, confirming their nanoscale dimensions, which are ideal for antibacterial and biological applications. The consistent size distribution and uniform morphology align with previous reports on biosynthesized ZnO NPs, which typically exhibit spherical or hexagonal shapes and sizes ranging from 18 to 46 nm [58–60]. Moreover, the close agreement between the particle size measured by TEM and the crystallite size calculated from XRD analysis (~ 33 nm) supports the structural integrity and homogeneity of the NPs. Owing to their high surface-area-to-volume ratio, these properties are particularly advantageous for enhancing surface reactivity, cellular interactions, and antibacterial efficacy. Overall, the TEM data demonstrate that the ZnO NPs successfully formulated a stable, nanoscale antimicrobial delivery system by maintaining desirable shape and dispersion.

**Fig. 6:**
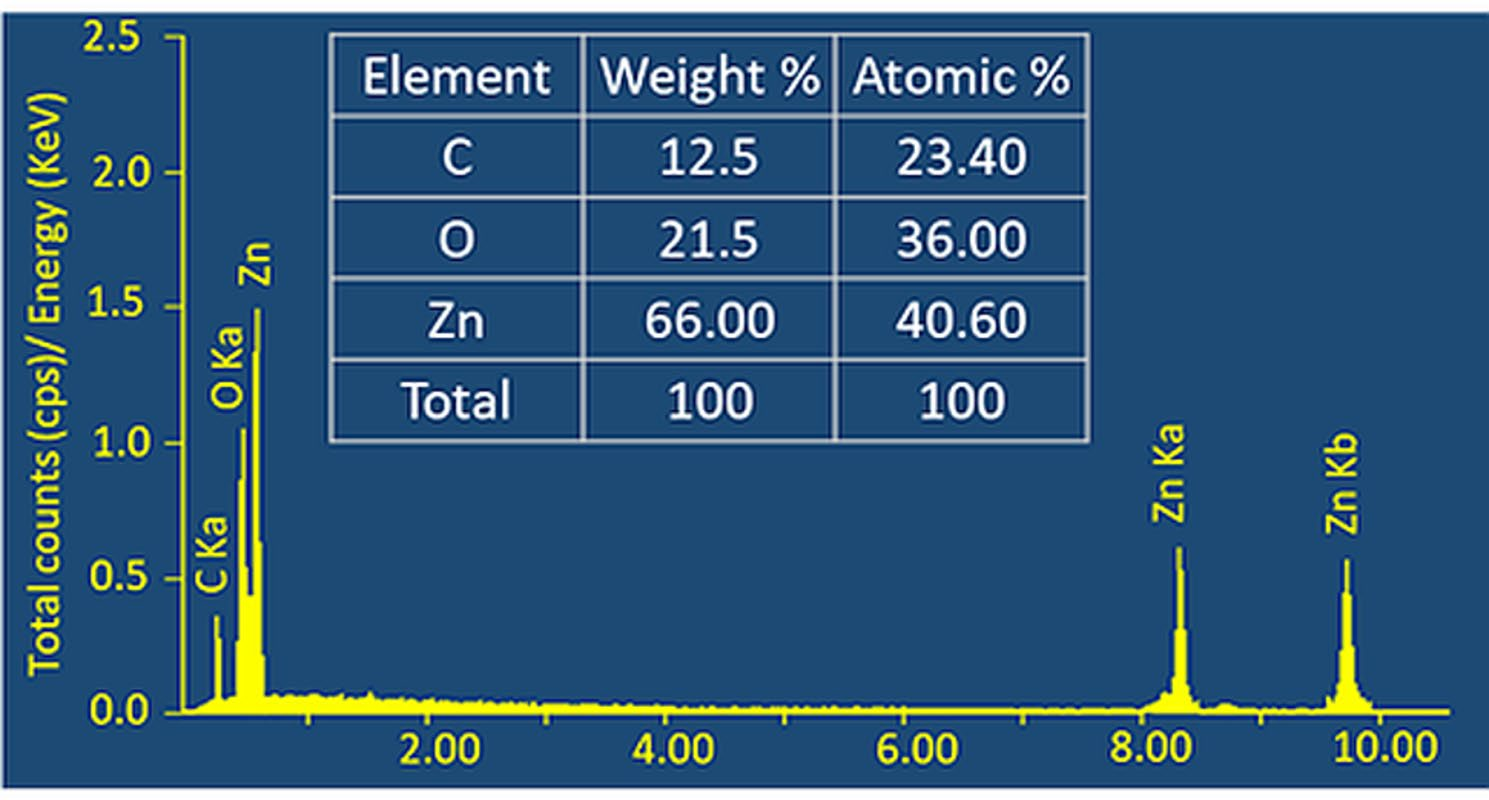
EDX spectrum and elemental analysis of ZnO NPs

The elemental composition of ZnO was analyzed using EDX, confirming the successful formation and purity of the ZnO material (Fig. 6). The EDX spectrum displays prominent and high-intensity peaks corresponding to Zn at 8.4 keV and 9.6 keV (Zn-K_a_ and Zn-K_b_ transitions, respectively) and oxygen at 0.5 keV (O-K_a_), which are characteristic signals of ZnO NPs. Quantitative analysis further revealed that the final product was mainly composed of Zn (66.00 wt%) and O (21.50 wt%), supporting the formation of a highly pure ZnO crystalline structure. Notably, a distinct carbon peak was detected at 0.3 keV, representing 12.50 wt% of the sample. The presence of carbon is expected and reflected the biogenic reduction and stabilization mechanism, confirming that organic residues from the *L. sordida* extract—such as proteins, polysaccharides, and other phytochemicals—remain adsorbed onto the surface of the ZnO NPs [32]. The absence of any additional elemental peaks in the EDX spectrum confirms that no extraneous impurities or toxic metal precursors were incorporated during the green synthesis process.

### GC–MS analysis of the ZnO NPs capped with *L. sordida* EGDA2 crude extract

**Fig. 7:**
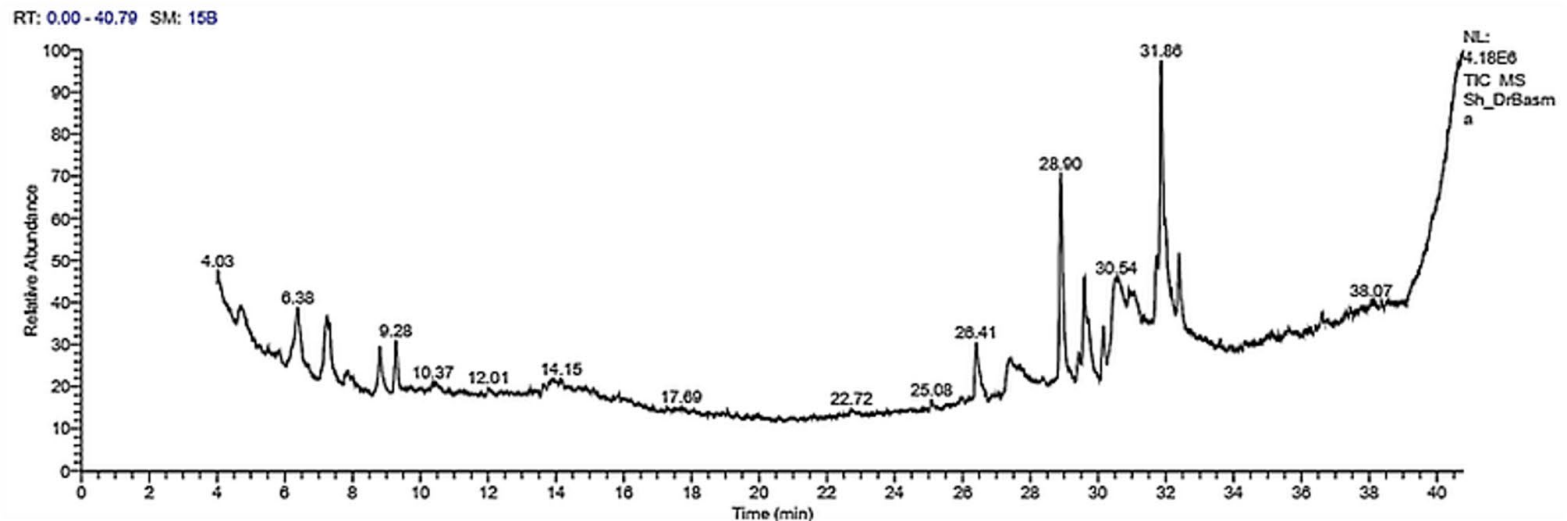
A GC-MS chromatogram of the ZnO NPs capped with *L. sordida* EGDA2 crude extract

Terpenoids, sesquiterpenes, exopolysaccharides, diatretol, lepistamides, and ketopiperazines are among the numerous secondary metabolites found in the genus *Lepista* (family Tricholomataceae), many of which possess strong antibacterial, antifungal, antimicrobial, antioxidant, and anti-inflammatory properties [61]. The ZnO NPs synthesized and capped with the *L. sordida* EGDA2 crude extract displayed a chemically diverse profile, primarily composed of polyphenols, fatty acids, and their derivatives, as revealed by GC–MS analysis. The chromatographic data showed several compounds, with nine prominent peaks at retention times (RT) 32.40, 31.86, 30.54, 28.90, 26.41, 9.28, 8.81, 7.24, and 6.38 min exhibiting a notably high abundance based on area percentage (Table 1; Fig. 7). These peaks represent the major constituents and suggest their significant contribution to the chemical properties of the nano-formulation. The compound eluting at RT 28.90 min (14.61%), one of the most prominent peaks in the GC-MS profile, was identified as hexadecanoic acid, trimethylsilyl ester (TMS derivative of palmitic acid), a saturated fatty acid with known antimicrobial, anti-inflammatory, and anti-tumor properties [62]. Its high abundance suggests a potential stabilizing role within the ZnO NPs formulation [63, 64]. The peak at RT 31.86 min (14.01%) was attributed to a mixture of long-chain hydrocarbon derivatives and fatty acid esters, including oleic acid and trimethylsilyl esters, which exhibit cytotoxic, membrane-disruptive, and antibacterial activities that may act synergistically to enhance the bioactivity of ZnO NPs [65]. Another significant peak at RT 32.40 min (4.77%) indicated the presence of unsaturated fatty acid esters, which are known to increase membrane fluidity and may facilitate cellular uptake and improve the delivery efficiency of the NPs system [66]. Furthermore, it was hypothesized that the compound eluting at RT 30.54 min (4.61%) was 9,12-octadecynoic acid, a TMS derivative, or a comparable acetylenic fatty acid. Such compounds are known to possess have cytotoxic, antimicrobial, and enzyme-inhibitory properties, potentially adding to the cytotoxic characteristics of the ZnO NPs [67–69]. Medium-chain fatty acid methyl esters, which are recognized for their antibacterial and surfactant-like properties, were identified as the source of the signal at RT 26.41 min (4.45%), possibly enhancing the dispersion and antimicrobial activity of the ZnO NPs [70, 71].

The preponderance of fatty acids and their esters in the GC–MS profile suggests that these compounds may serve a dual role: acting as natural capping agents to stabilize the ZnO NPs and enhancing their biological activity. Their well- documented antioxidant, cytotoxic, and antibacterial properties likely contribute synergistically to the observed bioactivity [22]. ZnO NPs/*L. sordida* EGDA2 represents a multifunctional antimicrobial platform with therapeutic significance and biological potential, as demonstrated by the complex interplay between its chemical constituents and the nanoparticulate carrier [12–15]. Based on these findings, this hybrid nanomaterial shows promise as a multi-mechanistic antibacterial agent capable of suppressing biofilms, induce oxidative stress, and disrupting membranes.

**Table 1:** Major compounds identified in the GC–MS profile of the ZnO NPs/*L. Sordida* EGDA2

RT (min)	Area (%)	Proposed compound identity	Compound class	Reported bioactivity	References
6.38	3.57	Hexadecanoic acid (TMS ester)	Saturated fatty acid derivative	Antioxidant, antibacterial, anti-inflammatory	[72–74]
7.24	4.77	α-Alanine	Amino acid	Essential for normal metabolism in humans	[75, 76]
8.81	4.24	d-Gala-l-ido-octonic amide (TMS derivative)	Octose carbohydrate derivative	Enzyme inhibition, cytotoxic, antimicrobial	[77]
9.28	4.28	Albuterol (TMS derivative)	Phenylethanolamine	Enhances lipophilicity and cellular permeability	[78, 79]
26.41	4.45	Medium-chain fatty acid methyl ester	Saturated fatty acid ester	Antimicrobial, surfactant activity	[80, 81]
28.90	14.61	Palmitic acid (TMS derivative)	Saturated fatty acid ester	Antimicrobial, surfactant activity, anti-inflammatory, anti-tumor	[82–85]
30.54	4.61	Octadecynoic acid (TMS derivative)	Acetylenic fatty acid	Enzyme inhibition, cytotoxic, antimicrobial	[86, 87]
31.86	14.01	Oleic acid/Linolenic acid ester (TMS/methyl)	Unsaturated fatty acid esters	Antibacterial, membrane disruptive, cytotoxic	[88–90]
32.40	4.77	Methyl linolenate or related PUFA ester	Polyunsaturated fatty acid	Anti-inflammatory, antioxidant, immune regulation	[86, 91]

### Antimicrobial activity

**Fig. 8:**
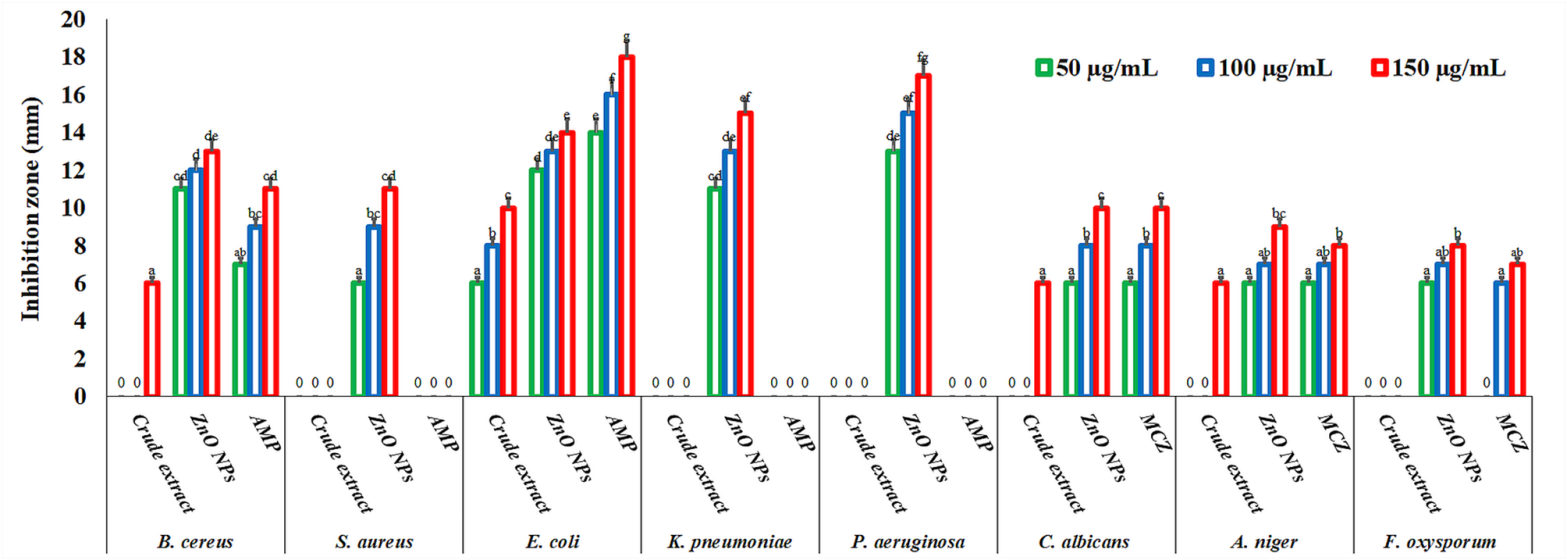
Antimicrobial activity of different concentrations from ZnO NPs (50, 100, and 150 µg/mL) using agar well diffusion method against the tested strains compared to crude extract of *L. sordida* EGDA2, ampicillin (AMP), and miconazole (MCZ). Means denoted by similar letter are not significantly different at *p* ≤ 0.05 using LSD test

The antimicrobial capabilities of ZnO NPs were evaluated using the agar well diffusion method, MIC, and MMC tests against a variety of Gram-positive and Gram-negative bacteria, yeast, and fungi, and compared to conventional drugs, AMP and MCZ. This study used the crude extract of *L. sordida* EGDA2 to create ZnO NPs, which were then increased their antimicrobial efficacy. The antimicrobial properties of ZnO NPs were evaluated using the agar well diffusion assay at different concentrations 50–150 µg/mL (Fig. 8). The findings revealed that ZnO NPs exhibited variable antimicrobial activity across the tested strains, producing significant inhibition zones. In agreement with earlier reports, the distinctly wider inhibition zones observed for ZnO NPs highlight their superior antibacterial efficacy [92–94]. Compared to Gram-positive bacteria, ZnO NPs were found to be more effective against the Gram-negative bacteria. Numerous parameters, such as the type of bacteria, the concentration of the antibacterial agent, the surface area, and the size and shape of NPs, all had an impact on the zone of inhibition (ZOI). The arrangement and composition of the cell membrane may also be connected to the variation in action against Gram-positive and Gram-negative bacteria [95]. This can be explained by significant alterations in the structure of bacterial cell walls. The thick peptidoglycan layer in Gram-positive bacteria may hinder the penetration of NPs, thereby limiting their interaction with intracellular components and reducing their antibacterial activity [96]. No inhibitory zones were observed against the resistant *Staphylococcus*, *Klebsiella* and *Pseudomonas* strains, suggesting no detectable antibacterial activity. Additionally, it was observed that MRSA, *K. pneumoniae*, *P. aeruginosa*, and *F. oxysporum* were not susceptible to the crude extract of *L. sordida* EGDA2. Only at high concentrations (150 µg/mL) did the crude extract show antimicrobial activity *B. cereus*, *C. albicans*, and *A. niger*. In contrast, the synthesized ZnO NPs demonstrated broad-spectrum antimicrobial activity against yeasts, fungi, and both Gram-positive and Gram-negative bacteria. The potential use of ZnO NPs as antimicrobial therapeutics in various industrial, medical, and environmental applications was confirmed by these findings.

**Fig. 9:**
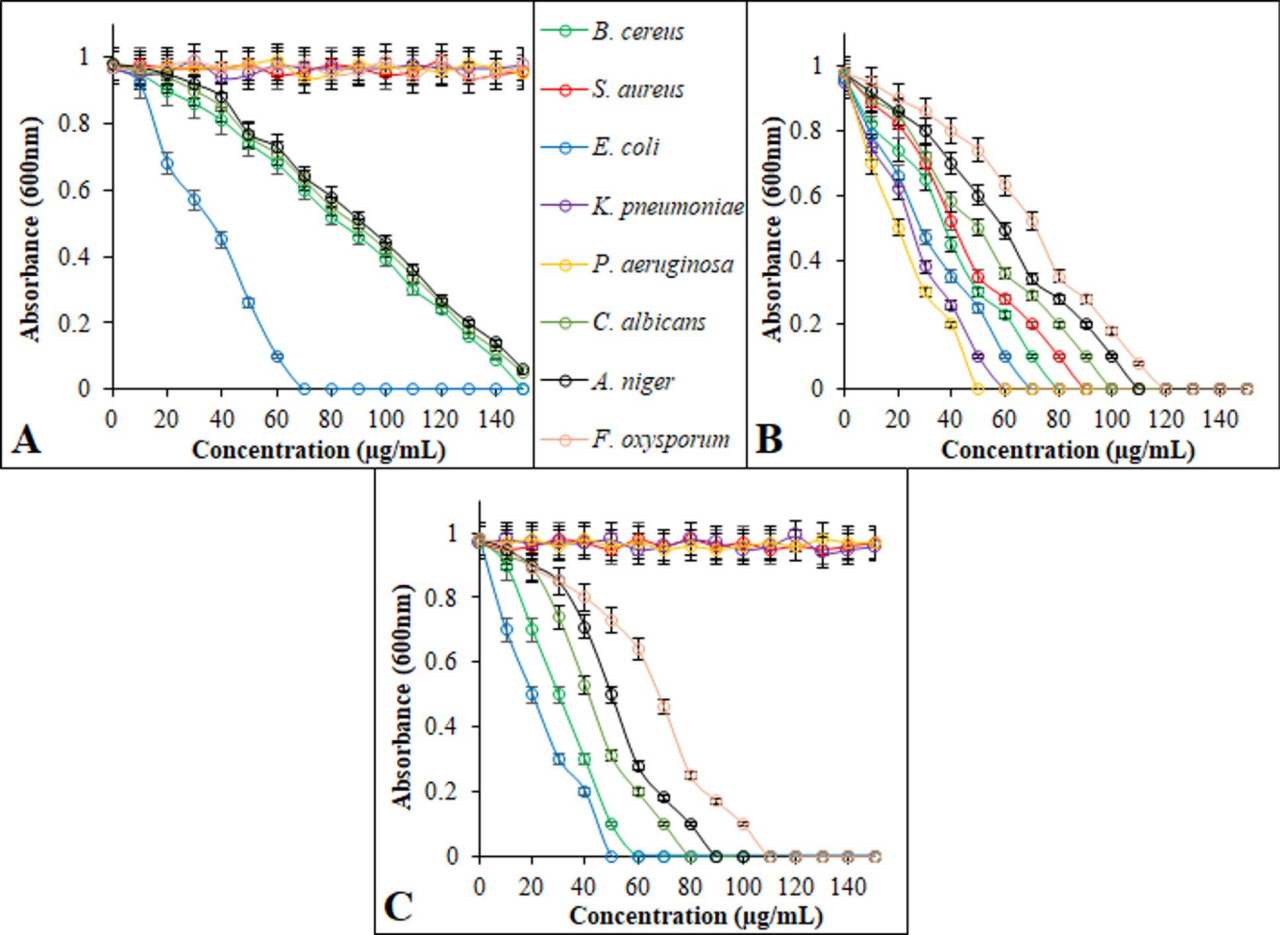
Minimum inhibition concentration of *L. sordida* EGDA2 crude extract; (**A**), ZnO NPs; (**B**), and standard antimicrobials; (**C**), against the tested microbial strains

**Fig. 10:**
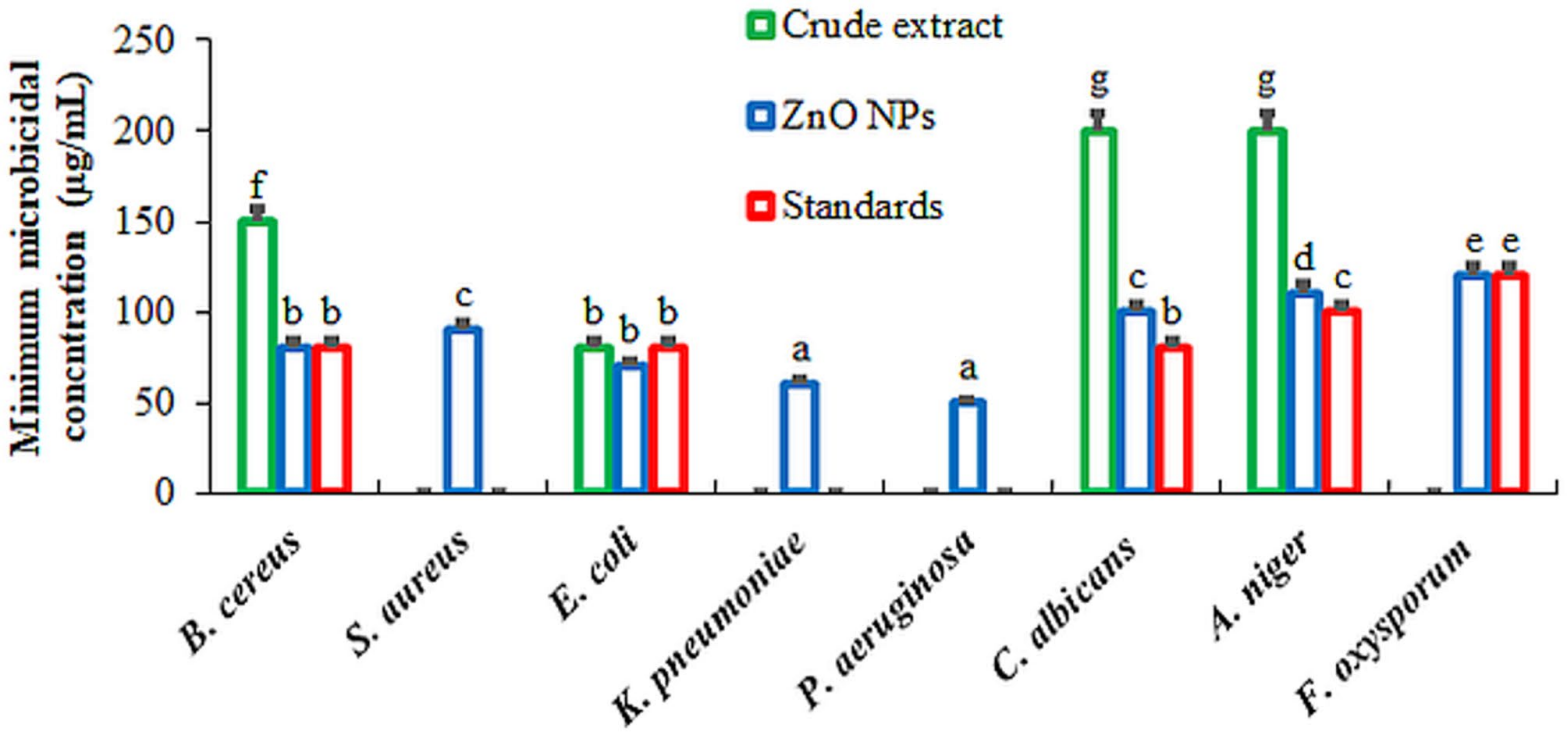
Minimum microbicidal concentration of *L. sordida* EGDA2 crude extract, ZnO NPs, and standard antimicrobials against the tested microbial strains.Means denoted by similar letter are not significantly different at *p* ≤ 0.05 using LSD test

The broth microdilution method was employed to determine the MIC of ZnO NPs within a concentration range of 1–150 µg/mL (Fig. 9). The ZnO NPs had the lowest MIC value, as anticipated, indicating the multi-component formulation’s increased efficacy. The MIC values ranged from 50 to 120 µg/mL for ZnO NPs, 70–150 µg/mL for *L. sordida* EGDA2 crude extract, and 50–150 µg/mL for conventional medications. The produced ZnO NPs showed much reduced MIC values that matched their MMC value (Fig. 10). The MMC values for ZnO NPs were 120 µg/mL to cause complete inhibition against all tested bacteria, yeast, and fungi. ZnO NPs have the best antimicrobial action against MRSA, *K. pneumoniae* and *P. aeruginosa* compared to the standard drug and *L. sordida* EGDA2 extract alone. Each component in the ZnO NPs/*L. sordida* EGDA2 has a distinct antibacterial effect. ZnO NPs induce oxidative stress and break down bacterial membranes by producing ROS [97]. While the bioactive compounds of *L. sordida* EGDA2 extract inhibit cell wall synthesis, support NPs retention, and increase permeability; ZnO NPs compromise bacterial membrane integrity and promote ROS generation; and although *L. sordida* EGDA2 extract is largely inactive alone, its inclusion may contribute to enhanced molecular interactions or intracellular delivery. This significant decrease in MIC reflects a synergistic antibacterial effect, where the distinct mechanisms of each component act in concert [98, 99]. The composite formulation successfully utilizes the complimentary activities of each element to overcome antimicrobial resistance and obtain higher efficacy at substantially lower dosages, as confirmed by the strong MIC reduction. These outcomes are in line with earlier research showing that multifactorial synergy in ZnO-based organic bioactive compounds led to better antimicrobial activity [100]. The combined formulation’s synergistic character, which required a significantly lower dose to prevent microbial growth, is confirmed by the MIC and MMC results. The improved efficacy in the present investigation is supported by these synergistic effects, which result in larger inhibitory zones and lower MICs as well as MMCs when compared to *L. sordida* EGDA2 extract alone. This finding is consistent with other research demonstrating that documented those combinations of ZnO NPs improve ROS generation, and microbicidal efficacy [101, 102].

### Antibiofilm investigations

Biofilms are bacterial communities that are organized and covered in a self-generated extracellular polymeric substance (EPS), which greatly increases their resistance to immune system defenses and conventional antibiotics [103]. The EPS matrix surrounds the biofilm cells and effectively protects them from a variety of bacteriostatic and bactericidal agents by preventing their penetration and adhesion to the bacterial cell walls, which increases the biofilm’s resistance to antimicrobial treatments [104]. ZnO NPs’ special qualities, such as their antibacterial activity and their capacity to disrupt biofilm architecture because of their small size and large surface area, make them a desirable choice for medical and industrial applications.

**Fig. 11:**
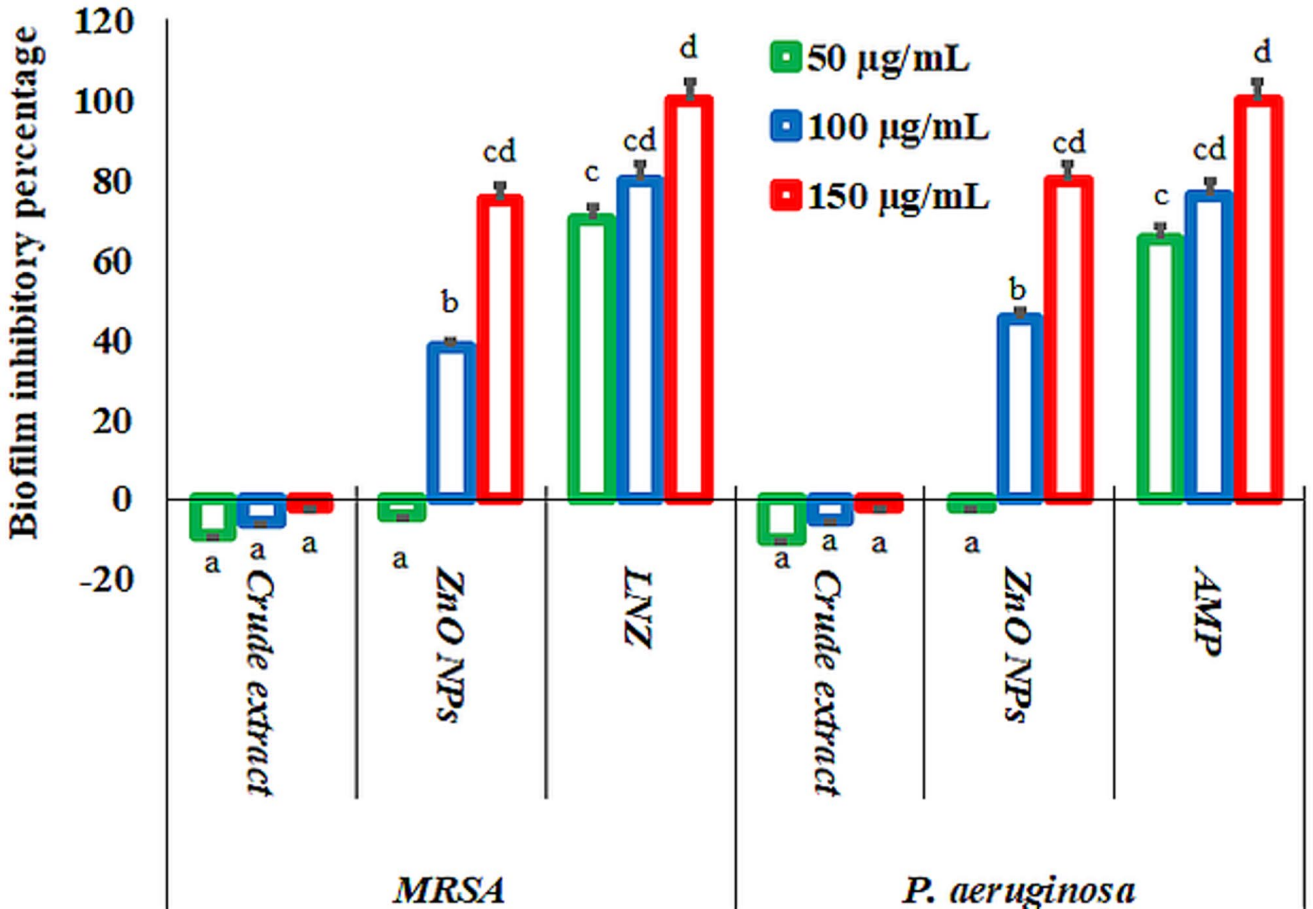
Antibiofilm test of ZnO NPs, linezolid (LNZ) and ampicillin (AMP) in different concentrations (50, 100, and 150 µg/mL) against biofilm-producing bacteria (MRSA, and *P. aeruginosa*). Means denoted by similar letter are not significantly different at *p* ≤ 0.05 using LSD test

In this study, the antagonistic potency of ZnO NPs capped with the bioactive compounds of *L. sordida* EGDA2 crude extract was investigated in inhibiting the formation of biofilms by biofilm-producing bacterial strains, including MRSA and *P. aeruginosa* (Fig. 11). The bacteria tested showed lower resistance to LNZ than to ZnO NPs, and the increase in the concentration of both LNZ and ZnO NPs enhanced biofilm inhibition. In the current study, ZnO NPs at their lowest concentration (50 µg/mL) were observed to influence bacterial metabolic activities and physiological functions [105]. The prepared nanomaterials exhibited greater efficacy against *P. aeruginosa* compared to MRSA. Slany et al. [106]. reported that exposing *S. aureus* to sub-inhibitory concentrations of methicillin or any other disinfectants can significantly induce biofilm formation by up-regulating the genes that encode surface proteins involved in this process. Additionally, Khiralla and El-Deeb [107] found that microelements were unable to eradicate the biofilms that was formed by *S. aureus* and *E. coli* at concentrations below 50 µg/mL. Husain et al. [108]. demonstrated that ZnO NPs inhibited Gram-negative biofilm growth up to 77% at a concentration of 128 µg/mL concentration. In a separate study, Husain et al. [109]. noted that 200 µg/mL of ZnO NPs was required to inhibit and promote the elimination of bacterial biofilms formation in *E. coli*, *S. aureus*, and *P. aeruginosa* by 62.80%, 71.57%, and 77.69%, respectively.

### CT-DNA binding investigation

**Fig. 12:**
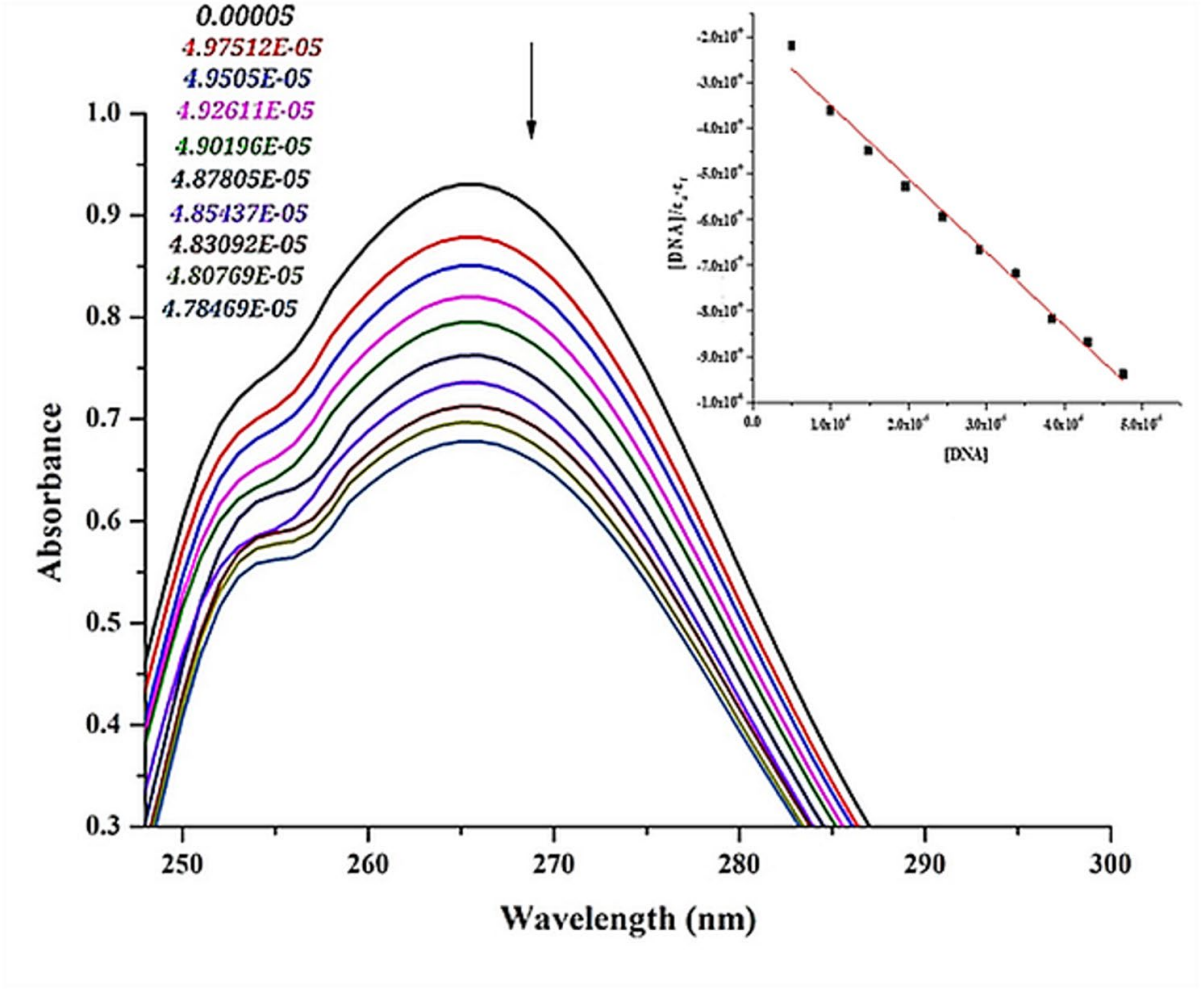
ZnO NPs absorption spectrum in a pH 7.2 buffer with the increasing CT-DNA concentration. The arrow illustrates how absorbance varies as CT-DNA concentration rises

The interaction of ZnO NPs with CT-DNA was quantified and investigated using electronic absorption spectroscopy (Fig. 12). CT-DNA experiments were performed at room temperature using specific concentrations of ZnO NPs, and the absorption spectra were measured to determine the intrinsic binding constant. The results showed that the absorption spectra decreased with increasing CT-DNA concentrations with ZnO NPs, exhibiting a slight hypochromic effect. The predicted K_b_ values for the ZnO NPs were 8.48 × 10^− 4^ M^− 1^, derived using the absorption spectrum method. Hypochromism was observed when the ZnO NPs intercalated with the calf thymus DNA. A possible explanation for this variation in band intensity is that the local relative orientation of the bases was perturbed to accommodate the ZnO NPs, which is necessary for the binding interaction between the NPs and CT-DNA. This observation is also consistent with the ZnO NPs’ higher selective binding affinity for CT-DNA. The interaction between ZnO NPs and CT-DNA is primarily non-covalent and can occur through intercalation, whereby the nanoparticles insert themselves between the base pairs of the DNA double helix. This binding mode is typically characterized by a marked decrease in DNA absorption intensity (hypochromism) and a shift in the absorption peak upon the addition of nanoparticles. Such intercalation can induce significant structural alterations in the DNA [110]. Additionally, ZnO NPs may bind within the major or minor grooves of the DNA helix. This groove-binding interaction is often electrostatic in nature, driven by the attraction between the negatively charged phosphate backbone of DNA and the positively charged surface of ZnO NPs, a process that can readily occur at neutral pH [111].

### Antioxidant assay

**Fig. 13:**
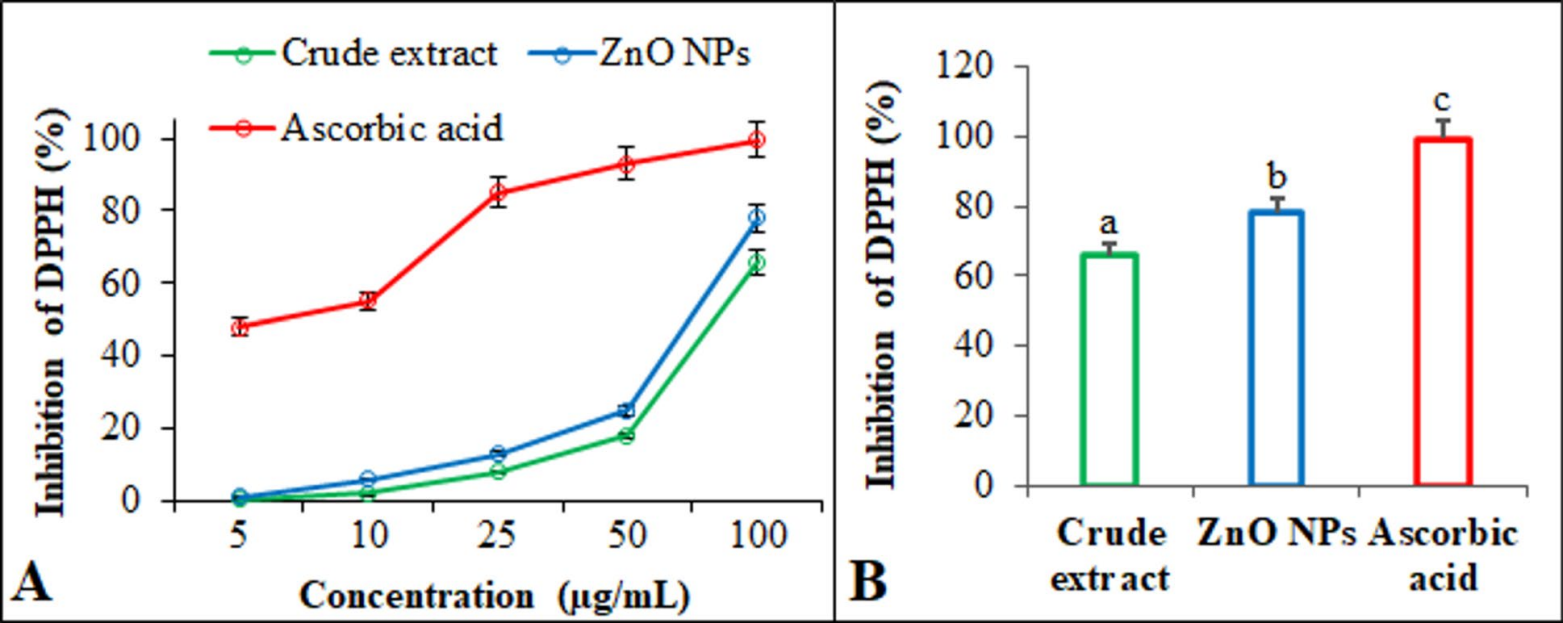
**A** DPPH scavenging activity of different concentrations the crude extract of *L. sordida* EGDA2, and ZnO NPs compared to the standard ascorbic acid. **B** Statistical comparison of the maximum percentage inhibition of the DPPH radical for the crude extract, ZnO NPs, and ascorbic acid at the highest tested concentration. Vertical bars represent the SD. Means denoted by similar letter are not significantly different at *p* ≤ 0.05 according to the LSD test

The in vitro DPPH radical scavenging method was employed to evaluate the antioxidant properties of the crude extracts of *L. sordida* EGDA2 and ZnO NPs (Fig. 13). The findings demonstrated that the crude extract of *L. sordida* EGDA2 had DPPH radical scavenging activity of 65.8%, while the ZnO NPs had 78.1%. Ascorbic acid, used as a standard antioxidant, demonstrated superior scavenging efficacy across all tested concentrations, consistent with previously reported findings [112, 113].

ZnO NPs—especially those synthesized via environmentally friendly (“green”) methods utilizing fungal or plant extracts—are capable of directly scavenging reactive species such as superoxide (O_2_^•−^), nitric oxide (NO^•^), and hydrogen peroxide (H_2_O_2_) [114]. This is often a result of the NPs’ high surface-to-volume ratio, which provides a large surface area for interaction with and neutralization of these harmful molecules.

ZnO NPs have demonstrated significant antioxidant properties, which are being explored for various biomedical and therapeutic applications. These nanomaterials have the potential to serve as a potent source for developing chemotherapeutic and therapeutic medicines for the treating pathological diseases caused by oxidative stress. Furthermore, the structure–activity relationship of the synthesized compounds is partly elucidated by their antibacterial, antifungal, antibiofilm, and antioxidant activities. Supporting analyses, including FTIR and GC–MS, confirm the presence of numerous bioactive compounds, which may account for the observed enhanced biological properties.

### Cytotoxicity assessment

**Fig. 14:**
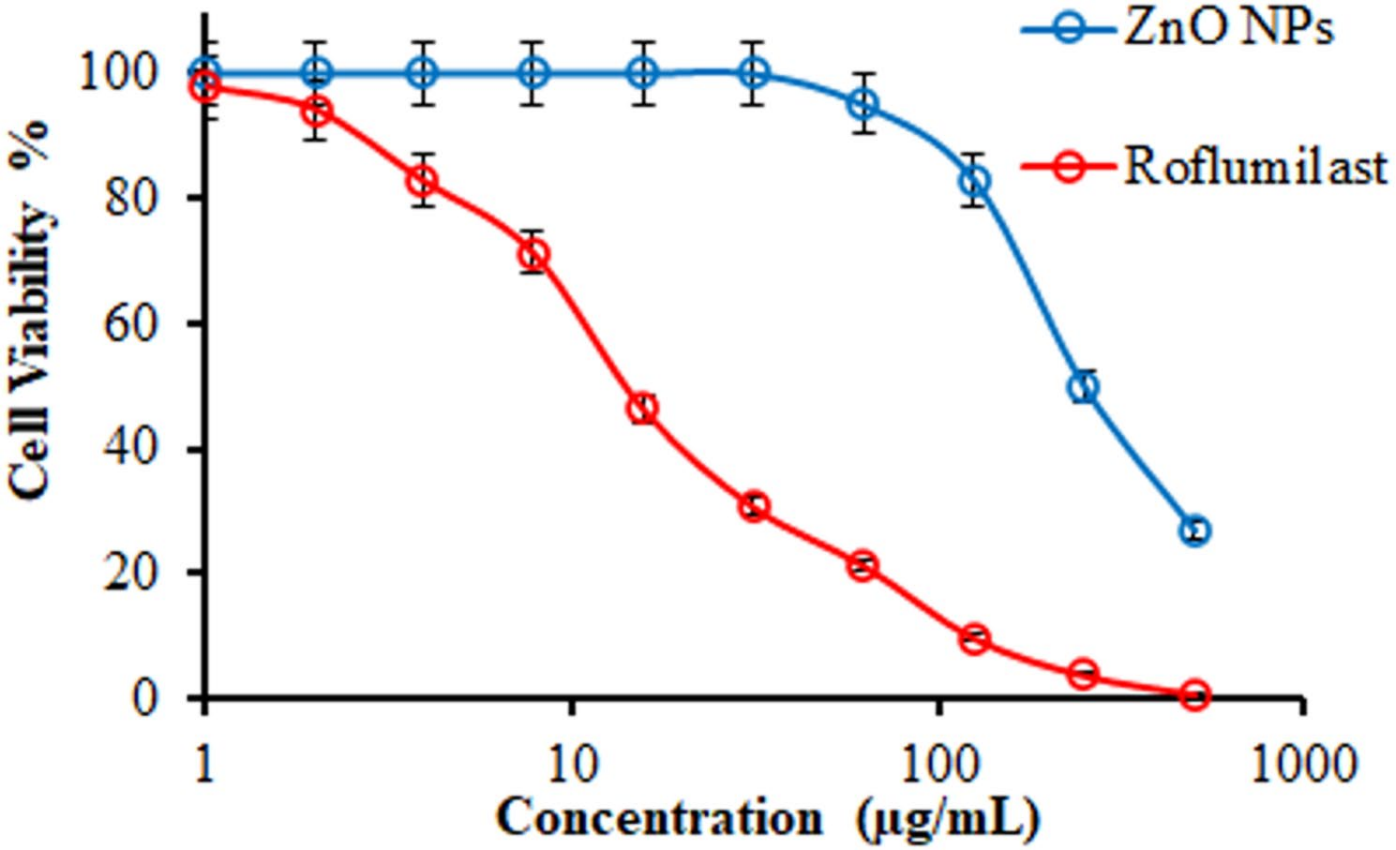
Cytotoxicity of ZnO NPs compared to the standard roflumilast against Vero cells

The cytotoxicity of green synthesized ZnO NPs was evaluated against the Vero cell line to determine their safety profile for biomedical applications (Fig. 14). The ZnO NPs demonstrated a high CC_50_ value of 208.17 ± 1.94 µg/mL, indicating significantly lower toxicity compared to the reference drug, roflumilast (CC_50_ = 15.13 ± 2.18 µg/mL). Notably, the CC_50_ of the ZnO NPs is substantially higher than the concentrations required for potent antimicrobial and antibiofilm activity (≤ 150 µg/mL), confirming a wide therapeutic window and a high degree of biocompatibility at effective dosages. These results corroborate previous studies highlighting that green-synthesized NPs are inherently safer and more environmentally benign than those produced via traditional chemical routes [14, 26, 110]. The safety profile was further evaluated by determining the selectivity index (SI) for each pathogen (Table 2). The SI, calculated as the ratio of CC_50_ to the maximum MMC (150 µg/mL), was > 1, ranging from 1.73 to 4.16, further supporting the preferential toxicity of these NPs toward microbial pathogens over mammalian cells. In contrast, the standard drug roflumilast exhibited a much narrower safety margin, with a CC_50_ nearly 14 times lower than that of the ZnO NPs. Furthermore, the absence of significant apoptotic effects at tested concentrations aligns with the findings by Fayed et al. [15], who reported that ZnO NPs typically maintain cellular integrity at concentrations below 31.25 µg/mL, further validating the potential of these biogenic ZnO NPs as safe candidates for future clinical nanomedicine applications.

**Table 2:** Selectivity index of ZnO NPs against pathogens

Target strain	CC_50_ (Vero Cells)	MBC Value (µg/mL)	SI
*B. cereus*	208.17 ± 1.94	80	2.60
MRSA	208.17 ± 1.94	90	2.31
*E. coli*	208.17 ± 1.94	70	2.97
*K. pneumoniae*	208.17 ± 1.94	60	3.47
*P. aeruginosa*	208.17 ± 1.94	50	4.16
*C. albicans*	208.17 ± 1.94	100	2.08
*A. niger*	208.17 ± 1.94	110	1.89
*F. oxysporum*	208.17 ± 1.94	120	1.73

The development of bioengineered nanomaterials presents revolutionary opportunities in nanomedicine, particularly for addressing the global crisis of antimicrobial resistance. Bioengineered NPs often exhibit synergistic or multimodal activity, enabling them to target pathogens through multiple mechanisms simultaneously (e.g., ROS generation, metal ion release, and interference with microbial biomolecules) [115]. The current findings, demonstrating combined antimicrobial, antibiofilm, and antioxidant activities, further highlight the multifunctional advantages of bioengineered nanomaterials. Green synthesis approaches, particularly those utilizing biological extracts such as *L. sordida*, are environmentally friendly, scalable, and minimize the use of hazardous chemicals, thereby offering a cost-effective pathway for large-scale production that is essential for future clinical translation. Moreover, the natural capping agents present in the biological extract serve as surface-functionalization molecules that enhance nanoparticle biocompatibility and stability, while also providing opportunities for subsequent modification for applications such as targeted drug delivery or bioimaging. Despite these promising attributes, the clinical translation of bioengineered NPs still faces major challenges, including comprehensive toxicity and biocompatibility assessment, scalability and batch-to-batch reproducibility, as well as navigating complex regulatory pathways [29, 116]. Although green synthesis generally produces NPs with lower toxicity, rigorous evaluation of cellular uptake, degradation pathways, and potential accumulation in sensitive organs remains essential, and accurately assessing long-term toxicity and in vivo biodistribution continues to be a major challenge [117]. Secondly, scaling up from laboratory synthesis to industrial production often introduces substantial variability; standardizing biological extracts and maintaining consistent particle size, morphology, and surface functionalization across large batches represents a significant technical challenge [118]. Finally, the lack of comprehensive, globally harmonized regulatory frameworks specifically tailored to complex, bioengineered nanomaterials slow clinical translation, highlighting the need for clear, nanomedicine-focused protocols for risk assessment and approval [119]. The current work, by demonstrating enhanced bioactivity through a green-engineered route, directly supports sustainability and efficacy goals, while explicitly acknowledging that further studies on toxicology, biodistribution, and process standardization are required before successful clinical and commercial implementation.

## Conclusions

A green synthesis of ZnO NPs was achieved using the wild mushroom *Lepista sordida* (Schumach) Singer EGDA2. Several physicochemical analyses, including UV-Vis spectroscopy, FTIR, Zeta potential, XRD, GC-MS spectrometry and SEM, were examined for the produced materials. The ZnO NPs’ zeta potential and FTIR spectrum show peaks at 2600–3300 cm⁻¹ and 979–1118 cm⁻¹ which could be stabilizing agents and suggest the particles’ long-term stability. Data from antimicrobial experiments employing the agar well diffusion technique, MIC, and MMC show that Gram-negative bacteria are more vulnerable to ZnO NPs than Gram-positive bacteria. The MIC values of the green-synthesized ZnO NPs showed robust microbicidal action against both Gram-positive and Gram-negative bacteria, ranging from 50 to 120 µg/mL. Furthermore, according to antiradical screening of the compounds against DPPH free radicals, ZnO NPs demonstrated the highest inhibition of DPPH radicals, with a %DPPH inhibition value of 78.1% compared to the crude extract of *L. sordida* EGDA2. ZnO NPs also prevented MRSA and *P. aeruginosa* from forming biofilms. Notably, their favorable interactions with DNA highlight promising pharmacological and biomedical applications. Furthermore, the safety of the green-synthesized ZnO NPs was empirically validated through cytotoxicity testing, where the nanoparticles exhibited a significantly higher CC_50_ (208.17 µg/mL) compared to the reference drug roflumilast (15.13 µg/mL). This wide therapeutic window confirms their potential as safe and effective candidates for future nanomedicine applications. Future in vivo studies on suitable animal models are warranted to further elucidate the antibacterial, antibiofilm, and toxicological mechanisms of ZnO NPs.

## Data Availability

The partial sequence of the ITS ribosomal RNA gene of *Lepista sordida* (Schumach) Singer strain EGDA2 obtained in the current study was deposited in the NCBI GenBank database under accession number: LN827702 and is available at the following URL: [https://www.ncbi.nlm.nih.gov/nuccore/LN827702] (https:/www.ncbi.nlm.nih.gov/nuccore/LN827702) The datasets generated during the current study are available from the corresponding author upon reasonable request.

## Abbreviations

AMP: Ampicillin
CT-DNA: Ca1f thymus DNA
CC_50_: 50% Cytotoxic concentration
DPPH: 2,2-diphenyl-1-picrylhydrazyl
EPS: Extracellular polymeric substance
FTIR: Fourier transform infrared spectroscopy
GC–MS: Gas chromatography–mass spectrometry
LNZ: Linezolid
MCZ: Miconazole
MIC: Minimum inhibition concentration
MMC: Minimum microbicidal concentration
SD: Standard deviation
SI: Selectivity index
TEM: Transmission electron microscopy
UV-Vis: Ultraviolet-visible spectroscopy
XRD: X-ray diffraction
ZnO NPs: Zinc oxide nanoparticles
